# The World Health Organization ACTION-I (Antenatal CorTicosteroids for Improving Outcomes in preterm Newborns) Trial: a multi-country, multi-centre, two-arm, parallel, double-blind, placebo-controlled, individually randomized trial of antenatal corticosteroids for women at risk of imminent birth in the early preterm period in hospitals in low-resource countries

**DOI:** 10.1186/s13063-019-3488-z

**Published:** 2019-08-16

**Authors:** WHO ACTION Trials Collaborators, Rajiv Bahl, A. Metin Gülmezoglu, My Huong Nguyen, Olufemi T. Oladapo, Gilda Piaggio, Joshua P. Vogel, Abdullah H. Baqui, Mohammod Shahidullah, Shivaprasad Goudar, Ashalata A. Mallapur, Shailaja Bidri, Sujata Misra, John Kinuthia, Zahida Qureshi, Frederick Were, Adejumoke Idowu Ayede, Bukola Fawole, Ebunoluwa A. Adejuyigbe, Oluwafemi Kuti, Shabina Ariff, Lumaan Sheikh, Sajid Soofi, James Neilson, Fernando Althabe, Harish Chellani, Elizabeth Molyneux, Kidza Mugerwa, Khalid Yunis, Liana Campodonico, Guillermo Carroli, Hugo Gamerro, Daniel Giordano, My Huong Nguyen, Janna Patterson, Abdullah H. Baqui, Rasheda Khanam, Meagan Harrison, Mohammod Shahidullah, Saleha Begum Chowdhury, Mohammad Abdul Mannan, Begum Nasrin, Salahuddin Ahmed, Nazma Begum, Saima Sultana, Soofia Khatoon, Anjuman Ara, Murshed Ahmed Chowdhury, Probhat Ranjan Dey, Dilip Kumar Bhowmik, Md. Abdus Sabur, Mohammad Tarek Azad, Gulshan Ara, Shaheen Akter, Sumia Bari, Md Mojibur Rahman, Farida Yasmin, M. A. Matin, Shahana Ferdous Choudhury, Shivaprasad S. Goudar, Sangappa M. Dhaded, Mrityunjay C. Metgud, Yeshita V. Pujar, Manjunath S. Somannavar, Sunil S. Vernekar, Veena Herekar, Vishwanath L. Machakanur, Shruti S. Andola, Ashalata A. Mallapur, Geetanjali M. Katageri, Sumangala Math, Bhuvaneshwari C. Yelamali, Ramesh Pol, Umesh Ramdurg, Shailaja R. Bidri, Sangamesh Mathpati, Preeti Patil, Bhavana B. Lakhkar, M. M. Patil, Muttu R. Gudadinni, Sujata S. Misra, Maya Padhi, Leena B. Das, Lucy Das, Saumya S. Nanda, Madhusmita J. Pradhan, Girija Shankar G. Mohanty, Rasmita S. Nayak, Bipsha S. Singh, Zahida Qureshi, Fredrick Were, Alfred Osoti, George Gwako, Ahmed Laving, John Kinuthia, Hafsa Mohamed, Faiza Nassir, Nayarit Mohamed, Adelaide Barassa, Joachim Ogindo, Bernard Gwer, Waweru Salome, Grace Ochieng, Njoroge John Githua, Bernadine Lusweti, Bukola Fawole, Adejumoke Idowu Ayede, Michael Abiola Okunlola, Olubukola A. Adesina, Adegoke Gbadegesin Falade, Oluwakemi Funmilola Ashubu, Olubunmi Busari, Wilfred Sanni, Aloysius Ebedi, Ejinkeonye I. Kate, Odiah Violet, Hadiza Abdulaziz Idris, Fatima Ali Sallau, Okoli Chinyere Viola, Ekwem Lilian Osaretin, Theresa Azonima Irinyenikan, Omolayo Adebukola Olubosede, Olufemi M. Omololu, Olugbenga Runsewe, Zainab Imam, Adesina Lawrence Akintan, Olorunfemi Oludele Owa, Olabanke Rosena Oluwafemi, Ireti Patricia Eniowo, Adetokunbo Fabamwo, Elizabeth Disu, Oluwafemi Kuti, Ibraheem Olayemi Awowole, Adebanjo Babalola Adeyemi, Akintunde Olusegun Fehintola, Ebunoluwa Aderonke Adejuyigbe, Henry Chineme Anyabolu, Bankole Peter Kuti, Olusola Comfort Famurewa, Adedapo Babatunde Anibaba Ande, Ikechukwu Okonkwo, Aboyeji Abiodun Peter, Mokuolu Olugbenga, Omotayo Adesiyun, Anthony Dennis Isah, Olateju Eyinade Kudirat, Olusanya Abiodun, Olabisi Florence Dedeke, Lawal Oyeneyin, Francis Bola Akinkunmi, Shabin Ariff, Lumaan Sheikh, Sajid Bashir Soofi, Nida Najimi, Mubarak Ali, Jamal Anwar, Saima Zulfiqar, Sadia Zulfiqar, Raheel Sikander, Shazia Rani, Salma Sheikh, Shazia Memon

**Affiliations:** World Health Organization, Geneva, Switzerland

## Abstract

**Background:**

Antenatal corticosteroids (ACS) have long been regarded as a cornerstone intervention in mitigating the adverse effects of a preterm birth. However, the safety and efficacy of ACS in hospitals in low-resource countries has not been established in an efficacy trial despite their widespread use. Findings of a large cluster-randomized trial in six low- and middle-income countries showed that efforts to scale up ACS use in low-resource settings can lead to harm. There is equipoise regarding the benefits and harms of ACS use in hospitals in low-resource countries. This randomized controlled trial aims to determine whether ACS are safe and efficacious when given to women at risk of imminent birth in the early preterm period, in hospitals in low-resource countries.

**Methods/design:**

The trial design is a parallel, two-arm, double-blind, individually randomized, placebo-controlled trial of ACS (dexamethasone) for women at risk of imminent preterm birth. The trial will recruit 6018 women in participating hospitals across five low-resource countries (Bangladesh, India, Kenya, Nigeria and Pakistan). The primary objectives are to compare the efficacy of dexamethasone with placebo on survival of the baby and maternal infectious morbidity. The primary outcomes are: 1) neonatal death (to 28 completed days of life); 2) any baby death (any stillbirth postrandomization or neonatal death); and 3) a composite outcome to assess possible maternal bacterial infections. The trial will recruit eligible, consenting pregnant women from 26 weeks 0 days to 33 weeks 6 days gestation with confirmed live fetuses, in whom birth is planned or expected within 48 h. The intervention comprises a regimen of intramuscular dexamethasone sodium phosphate. The comparison is an identical placebo regimen (normal saline). A total of 6018 women will be recruited to detect a reduction of 15% or more in neonatal deaths in a two-sided 5% significance test with 90% power (including 10% loss to follow-up).

**Discussion:**

Findings of this trial will guide clinicians, programme managers and policymakers on the safety and efficacy of ACS in hospitals in low-resource countries. The trial findings will inform updating of the World Health Organization’s global recommendations on ACS use.

**Trial registration:**

ACTRN12617000476336. Registered on 31 March 2017.

**Electronic supplementary material:**

The online version of this article (10.1186/s13063-019-3488-z) contains supplementary material, which is available to authorized users.

## Background

### The global burden of preterm birth

An estimated 14.84 million babies were born preterm in 2014, accounting for 10.6% of all live births worldwide [1]. Complications of preterm birth were the leading cause of death in children under 5 years of age globally in 2016, accounting for approximately 16% of all deaths, and 35% of deaths among newborn babies [2]. Preterm neonates are at increased risk of a wide range of short- and long-term respiratory, infectious, metabolic and neurological morbidities, with higher risks of adverse outcomes seen at lower gestational ages [3, 4]. Notably, infants born prior to 34 weeks have significantly worse morbidity and mortality outcomes compared with late preterm infants (34 to < 37 weeks) [3, 4], including higher rates of respiratory distress syndrome, bronchopulmonary dysplasia, necrotizing enterocolitis, intraventricular haemorrhage and infections [5–9]. Infants born preterm experience more hospital readmissions [10, 11], as well as higher rates of neurodevelopmental disorders, impairments of cognitive functioning, behavioural problems, psychiatric disorders, attention-deficit hyperactivity disorder and poorer academic achievement [12–16]. Preterm birth and its sequelae can have negative psychosocial and financial impacts on families of preterm newborn babies [9, 17–19].

### Antenatal corticosteroids

Dexamethasone is a synthetic, anti-inflammatory 9-fluoro-glucocorticoid. It is one of the most active glucocorticoids, being about 25 to 30 times more potent than hydrocortisone, and exerts effects solely via the glucocorticosteroid receptor [20]. Animal and human models have shown that glucocorticoids enhance the structural maturity of developing fetal lungs, including differentiating mesenchymal tissue, accelerating production and secretion of surfactant and decreasing vascular permeability, leading to increased compliance and maximal lung volume [21]. It is in routine clinical use for a variety of health conditions, and is widely used in women at risk of imminent preterm birth to prevent morbidity and mortality in preterm newborn babies.

The first randomized controlled trial of antenatal corticosteroids (ACS; betamethasone) in humans to prevent respiratory distress syndrome was published in 1972 [22]. The Cochrane systematic review on ACS for accelerating fetal lung maturation for women at risk of preterm birth includes 30 trials of 7774 women and 8158 infants [23]. While the review shows significant reductions in neonatal mortality and several morbidities associated with the use of ACS, a close examination of this evidence base reveals several limitations, particularly regarding its generalizability to lower-resource countries. We recently published an article detailing the limitations of the current evidence base on ACS use in these settings including why the World Health Organization (WHO) ACTION (Antenatal CorTicosteroids for Improving Outcomes in preterm Newborns) trials are needed [24]. In brief, previous ACS efficacy trials have been largely conducted in tertiary hospitals in high-income countries. The 30 trials were conducted in higher-level hospital settings, in high-income (20 trials) and upper middle-income (nine trials) countries, except one trial that was conducted in Tunisia (a lower middle-income country). Trials have recruited heterogeneous or highly selected populations of women, and no trial has been independently powered for neonatal mortality. The Cochrane review authors and other researchers have cautioned that, while this evidence is conclusive for hospital settings in higher-income countries, its generalizability to lower-income countries (where the majority of the world’s births occur) is limited [23, 25].

Concerns regarding ACS safety and efficacy in low-resource settings were recently raised by the adverse findings of the Antenatal Corticosteroids Trial (ACT) [26]. ACT was a community-based, cluster-randomized implementation trial conducted in six low- and middle-income countries (Argentina, Guatemala, India, Kenya, Pakistan and Zambia). The trial evaluated a multifaceted intervention designed to increase the use of ACS at all levels of the healthcare system. Trial outcomes included stillbirth, neonatal mortality and suspected maternal infections. Among the less-than-5th-percentile newborn babies (a proxy for preterm births), ACS use did not affect the rate of neonatal deaths before 28 days (relative risk (RR) 0.96, 95% confidence interval (CI) 0.87–1.06, *p* = 0.65). Among all births, the risk of perinatal deaths increased (RR 1.11, 95% CI 1.04–1.19), driven by increases in both the risk of neonatal mortality by 28 days (RR 1.12, 95% CI 1.02–1.22) and of stillbirth (RR 1.11, 95% CI 1.02–1.22). Furthermore, the intervention was associated with increased odds of suspected maternal infection in women with births with less-than-5th-percentile (10% vs 6%, odds ratio (OR) 1.67, 95% CI 1.33–2.09) and women overall (3% vs 2%, OR 1.45, 95% CI 1.33–1.58). The ACT findings have raised concerns that ACS use in peripheral levels of the healthcare system in lower-income countries may confer no benefit and are harmful. However, there is currently no trial evidence to guide clinicians as to whether ACS is efficacious or safe in reasonably equipped hospitals in low-resource countries.

### WHO recommendations on preterm birth (2015)

WHO currently recommends that ACS be used for women at risk of preterm birth from 24 weeks to 34 weeks gestation when the following five criteria are met [27]: 1) gestational age assessment can be accurately undertaken; 2) preterm birth is considered imminent; 3) there is no clinical evidence of maternal infection; 4) adequate childbirth care is available (including the capacity to recognize and safely manage preterm labour and birth); and 5) the preterm newborn baby can receive adequate care if needed (including resuscitation, thermal care, feeding support, infection treatment and safe oxygen use).

WHO recommends that either dexamethasone or betamethasone can be used, although it is noted that dexamethasone is more widely available, at lower cost, and currently listed on the WHO Essential Medicines List [28]. The ACS treatment criteria were consensus-based and intended to address concerns regarding ACS safety in resource-limited settings. The recommendation remarks specify that, based on current evidence, ACS should not be routinely administered where these criteria are not met as the risk of neonatal harm may outweigh the benefits if matured babies are exposed to corticosteroid in utero [29]. Both the guideline panel and a subsequent WHO consultation of independent obstetric and neonatal experts identified that further ACS trials are needed in hospitals in lower-income countries to determine whether they can be used safely and efficaciously [24].

## Methods

### Aims and objectives

The aim of this trial is to determine whether ACS are safe and efficacious for women and newborn babies in hospitals in resource-limited settings when given to women with live fetuses at risk of imminent preterm birth between 26 weeks 0 days and 33 weeks 6 days gestation for the prevention of neonatal death. The primary objectives are to compare the effect of dexamethasone with placebo on stillbirth, neonatal survival and possible maternal bacterial infections when given to women at risk of imminent preterm birth in participating hospitals.

### Hypotheses

We hypothesised that ACS use will result in clinical benefits for the baby without increasing the risk of harm to the mother. Therefore, we will apply a superiority hypothesis for the primary outcomes relating to neonatal death and any baby death, and a noninferiority hypothesis for the outcomes relating to maternal infection.

### Type of trial

The design is a multi-country, multi-centre, parallel, two-arm, double-blind, placebo-controlled trial of ACS for women at imminent risk of birth in the early preterm period. Women will be individually randomized (in a 1:1 ratio) to dexamethasone or placebo (normal saline). An individual randomization design was chosen as the intervention is at the level of the individual woman. The trial will test the superiority hypotheses regarding baby outcomes, and the noninferiority hypothesis regarding maternal outcomes, to 28 completed days after birth. The trial will be conducted in compliance with the trial protocol and good clinical practice (GCP) standards. This study protocol was developed in accordance with the Standard Protocol Items: Recommendations for Interventional Trials (SPIRIT) guidance [30]. See Additional file 1 for the SPIRIT checklist.

### Study setting

The trial will be conducted in participating hospitals across six study sites in five countries—Bangladesh, India, Kenya, Nigeria (Ibadan), Nigeria (Ile-Ife) and Pakistan. Participating hospitals were selected through a standard assessment of available interventions and quality of care. The emphasis was on identifying hospitals in lower-income countries where a minimum standard of maternal and newborn baby care for preterm birth can be provided according to the WHO ACS treatment criteria [29]. Specifically, hospitals where adequate childbirth care is available (including the capacity to recognize and safely manage preterm labour and birth), and where preterm newborn babies can receive adequate care if needed (including resuscitation, thermal care, feeding support, infection treatment and safe oxygen use). Hospital selection was also based on adequate preterm birth rate and minimal out-referral (to optimize recruitment and follow-up of trial participants). These hospitals do, however, experience the human resource, referral and health service equipment challenges characteristic of low-resource countries.

### Participants

The populations of interest are: a) pregnant women (with confirmed live fetuses) from 26 weeks 0 days to 33 weeks 6 days gestation in whom birth is planned or expected within 48 h; and b) their fetuses and newborn babies. The inclusion criteria are: 1) birth planned or expected within 48 h^1^; 2) gestational age from 26 weeks 0 days to 33 weeks 6 days^2^ informed by ultrasound; 3) women with singleton or multiple pregnancies, where the fetus(es) is(are) alive; 4) women with no clinical signs of severe infection (as per clinical assessment)^3^; and 5) women willing and able to provide informed consent.

Women with a history of previous preterm birth, hypertensive disorders, a growth-impaired fetus, diabetes or HIV are eligible; comorbid conditions will be managed according to local guidelines.

The exclusion criteria are: 1) intrauterine fetal death; 2) identified major or lethal congenital fetal anomaly; 3) no prior ultrasound-based estimate of gestational age available and immediate ultrasound examination is not possible; 4) any concurrent or recent (within the past 2 weeks) systemic corticosteroid use during the current pregnancy (outside of the trial); 5) currently a participant in another clinical trial related to maternal and neonatal health; and 6) any other clinical indication where the treating clinician considers corticosteroids to be contraindicated.

A subset of participants may not give birth within 7 days of randomization. These women will be reassessed (upon representation to study hospital) for use of a repeat course. A repeat course will be used if 7 completed days have elapsed since the first dose of the first course, birth is planned or expected within the next 48 h, and the woman is still eligible according to the above criteria. In the event a woman with a multiple pregnancy requires a repeat course, the repeat course will be used if at least one fetus is alive.

### Intervention and control

WHO currently recommends that either dexamethasone or betamethasone (total 24 mg in divided doses) can be used for this indication; however, this trial will use dexamethasone on account of its wider availability, lower cost, and listing on the WHO Essential Medicines List. Dexamethasone sodium phosphate for intramuscular injection (4 mg/1 mL) will be used. The trial regimen is in line with WHO recommendations, namely: 1) a single dose of 6 mg intramuscular dexamethasone administered every 12 h, to a total of four doses (time points 0 h, 12 h, 24 h and 36 h) until course completion, discharge or birth (whichever comes first). If the full course is completed, the woman will receive a total of 24 mg in four divided doses; 2) if a woman has not delivered within 7 completed days after the first dose, is reassessed as eligible, and a subsequent clinical assessment demonstrates that birth is planned or expected in the next 48 h, a second course (repeat treatment pack) according to the regimen described above will be administered. The repeat course is an identical regimen to the first course, and is the same as the initial allocation (i.e. women initially randomized to dexamethasone will receive dexamethasone as repeat course and women initially randomized to placebo will receive placebo); and 3) a woman may have a maximum of two full courses only—no subsequent courses will be used even if delivery does not occur as expected or planned.

The control intervention is an identical placebo of normal saline, administered according to the same regimen.

### Outcomes

This trial has three primary outcomes: 1) neonatal death (death of a live birth within 28 completed days of life); 2) any baby death (any death of a fetus (postrandomization) or death of a live birth within 28 completed days of life among all randomized participants); and 3) possible maternal bacterial infection (occurrence of maternal fever or a clinically suspected or confirmed infection for which therapeutic antibiotics were used).

Secondary outcomes include a range of maternal and newborn baby morbidity and mortality outcomes, as well as health service utilization outcomes. All outcomes and operational definitions are provided in Additional file 2.

### Participant timeline

**Fig. 1:**
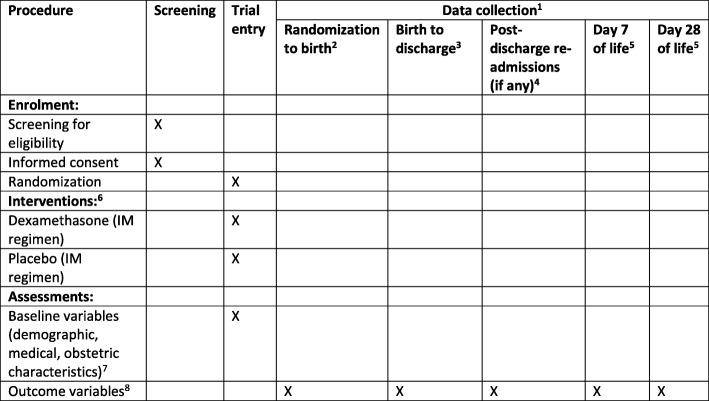
SPIRIT figure for the Antenatal Corticosteroids for Improving Outcomes in preterm Newborns (ACTION)-I trial. ^1^ Data will be collected from randomized women from time of randomization to day 28 postpartum. Data will be collected on all newborn babies (single or multiple) from time of birth to day 28 postnatal or death. ^2^ Data will be collected from time of randomization. Some randomized women may be discharged without giving birth; however, data collection continues when they are readmitted later in the pregnancy for birth. ^3^ Data will be collected for women and newborn babies during admission until discharge. If the length of admission exceeds 28 days, data will be collected to 28 completed days only. ^4^ Women or newborn babies who experience a readmission to hospital during the follow-up period (postdischarge from hospital following birth) will have data collected. ^5^ Day 7 and day 28 follow-up visits will be performed, regardless of location (hospital or community). ^6^ The regimen is described in the study protocol. A full course (four doses) takes a total of 36 h to administer. In the event that a randomized woman does not give birth within 7 days, she may be eligible for a repeat course (four doses). ^7^ Baseline variables are collected after the first dose has been administered. Baseline variables include: age, education, marital status, gravidity, parity, maternal history of preterm birth, weight, height, mid-upper arm circumference, medical conditions (chronic hypertension, diabetes mellitus, HIV/AIDS, tuberculosis, pyelonephritis, anaemia, malaria), obstetric conditions (gestational diabetes, preterm prelabour rupture of membranes, pre-eclampsia or eclampsia, gestational hypertension, oligohydramnios, polyhydramnios, intrauterine growth restriction (known or suspected), macrosomia, abruptio placentae, placenta praevia, other obstetric haemorrhage), gestational, use of tocolysis, symptoms of imminent preterm birth. ^8^ All outcome variables are described in Additional file 2. IM intramuscular

The participant timeline and follow-up process is summarized in Fig. 1. Screening, informed consent and randomization will take place in study hospitals. Trial participants are both women and their babies, and will be followed-up from randomization to 28 completed days after birth, regardless of location (hospital or community). Participants will be asked for contact information to facilitate follow-up, and will be advised to return to the study hospital in the event of any adverse outcomes for her or her baby. Scheduled follow-up visits will be conducted around day 7 and day 28 postpartum/postnatal.

### Sample size

Sample size is calculated on the basis of the primary outcome of neonatal mortality at 28 completed days. A total of about 5416 women are needed to detect a reduction of 15% or more from 25% deaths to 21.3% among neonates from women who were administered ACS at < 34 weeks in a two-sided 5% significant test with 90% power. With 10% loss to follow-up, about 6018 women will need to be recruited.

Sample size has also been determined separately for the maternal infection primary outcome as a noninferiority hypothesis is required. A noninferiority hypothesis considers that the intervention is no worse than the comparator by more than a prespecified minimum difference (Δ). The prevalence of possible maternal bacterial infection for this trial is estimated at 10%, based on an assessment of rates of maternal infection-related outcomes in previous ACS efficacy trials [23]. The critical margin of noninferiority selected for this outcome is 2.5%, which represents a 25% difference from the baseline prevalence (10%) and is regarded as clinically relevant. Hence, under a noninferiority hypothesis a total sample size of 5024 women are needed (including 10% loss to follow-up) to demonstrate noninferiority within the 2.5% margin for the maternal infection outcome, assuming equal prevalence of 10% in the two arms, with a power of 80% and a significance level of 2.5%. It is important to note the relative importance of the three primary outcomes. If noninferiority of dexamethasone with respect to placebo is not demonstrated for possible maternal infection, this will need to be weighed against the effects (potential benefit) seen for the other two primary outcomes, which may be considered as having greater clinical significance.

### Screening, consent and recruitment to the trial

Women will be first routinely evaluated on arrival at participating hospitals by obstetric care providers presenting to the antenatal ward or labour ward admission area (or other similar clinical areas). In those women with clinical features or indications suggestive of imminent preterm birth, study staff (including research or clinical staff trained in study procedures) will conduct formal screening using a standardized screening form. Women may be referred for an ultrasound for gestational age assessment as part of this screening process (if an obstetric ultrasound of reasonable quality for gestational age estimation has not been performed previously during the current pregnancy). Women who are experiencing labour symptoms will only be approached for screening if their vital signs are normal, and they are not unduly stressed (signs indicating stress include tachycardia and tachypnea, as well as presenting in general state of distress). If women are unable to complete the full screening and informed consent process (due to pain, obstetric complications or other reasons) they will not be recruited. Each centre will screen and recruit potential participants until the per-centre sample size is achieved.

For women meeting the eligibility criteria, an informed consent process will be conducted (Additional file 3). All women will receive information about the trial in their language of choice via an information sheet. Participants will be given time to reflect on the information and given an opportunity to ask questions. If willing to participate, the informed consent form will be signed by the participant and study staff. For minors, informed assent and parental consent forms may be used (in accordance with local Institutional Review Board (IRB) guidance). Participants will be free to withdraw from the trial at any stage without loss of benefits. Women who do not wish to participate are free to say so, and will receive the same level of care they would normally receive. If a woman is nonliterate, an impartial witness will be present during the entire informed consent reading and discussion, and will also sign the form. The contact information of the investigators will be made available to participants in the event that they require further information or assistance. There will be no payment for participation; however, some sites will offer reimbursement for transport costs incurred during follow-up visits. Clinicians in participating hospitals will be informed about the trial. Enrolled participants will be asked to complete a contact information form to facilitate follow-up, and will be provided with a trial card. Research teams will use short message service (SMS) reminders to advise participants of forthcoming appointments.

### Allocation, sequence generation, concealment and blinding

**Fig. 2:**
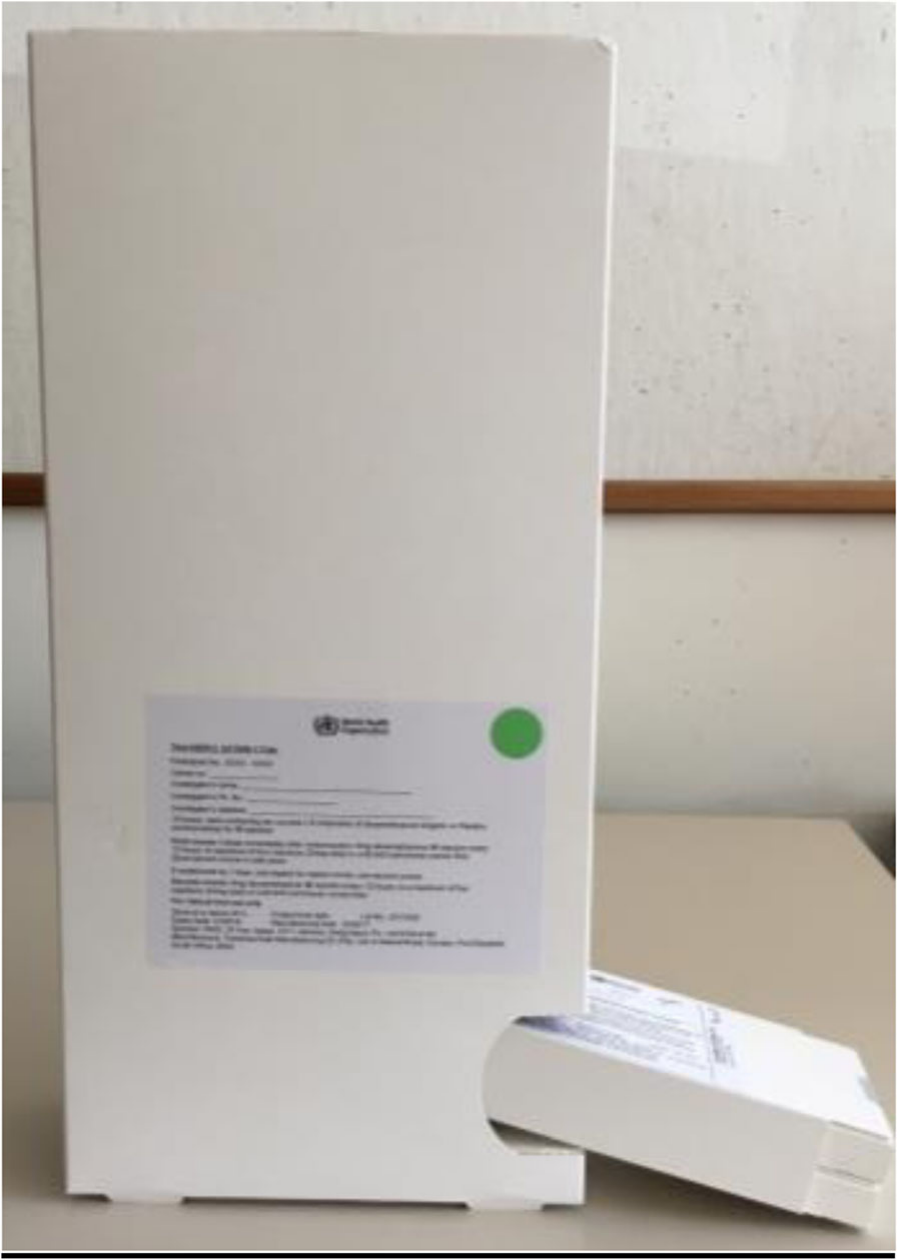
Study boxes and dispensers

In this trial, site-stratified individual randomization will be generated centrally at WHO Headquarters. Participants will be randomly assigned to either control or experimental groups (allocation ratio of 1:1) as per a computer-generated randomization sequence in permuted blocks. All sites will receive identical treatment packs containing sufficient ampoules of dexamethasone or placebo for two full courses (in the event both initial and repeat treatments are needed) according to the randomization sequence, assembled consecutively in special dispensers (see Fig. 2). The assignment schedule will be stored at WHO.

Once eligibility is confirmed and consent obtained, trained study staff will randomize a woman by taking the next numbered treatment pack from the dispenser (which is designed to ensure packs are taken sequentially) (Fig. 2). A study pack contains sufficient ampoules for two full courses (in the event that both initial and repeat treatment are needed). The unique participant number is on the study pack (as a detachable sticker). Those women who complete the first course and have not given birth by 7 days will be reassessed for eligibility for a repeat course (which is the same as the initial allocation). Participants, care providers, study staff and outcome assessors will be blinded to the group allocation. Sealed code-break envelopes that contain each participant’s treatment allocation will be securely stored at the site for emergency unblinding only in the rare event that emergency unblinding is needed. All emergency code-break envelopes will be returned to WHO at the end of the trial.

### Data collection and management

Outcomes of interest are clinical and health service utilization outcomes. Data will be collected on standard paper forms, according to the trial Manual of Operations. Designated forms are available (if required) for adverse events, serious adverse events, protocol deviations or protocol violations. Data forms will also collect information on how many injections have been administered. Collected data will then be double-entered (at hospital or site level) into a web-based, GCP-compliant data management platform (Kamolo, Centro Rosarino Estudios Perinatales (CREP), Argentina), overseen by the site data managers. All data will be managed centrally by a trial data management team (CREP, Rosario, Argentina, and WHO, Geneva, Switzerland). A validation system has been built into the data management system to ensure consistency, accuracy and completeness of the data collected.

All study-related information will be stored securely at the study site. All participant information will be stored in locked file cabinets in secure rooms accessible only by designated study staff. All records that contain names or other personal identifiers will be stored separately from study records identified by participant number. All local databases will be secured with password protection. Participants’ study information will not be released outside of the study without the written permission of the participant.

### Statistical methods and analysis

The study statistician will be responsible for overseeing data management, as well as development of the statistical analysis plan and execution of the preplanned analyses (and any subsequent secondary analyses). The first version of the statistical analysis plan including dummy tables was finalized before recruitment began. The final statistical analysis plan will be agreed by the investigator group prior to completion of the trial and unblinding of participants.

For women, the intention-to-treat (ITT) population will be defined as all randomized women according to treatment assignment, regardless of compliance, excluding those withdrawing consent after randomization. However, if consent withdrawal is only for continuing to participate in the study, but not for using the data, then collected data for these participants will be included in the analysis. The per-protocol (PP) population will be all women in the ITT population excluding those with protocol violations that might affect the primary outcomes. For babies, similar populations will be defined according to their mothers.

The main analysis will be based on the ITT population, analysing all participants with outcome data available. Analysis of primary outcomes will be corrected for multiplicity (i.e. multiple primary outcomes). The primary outcomes of any baby death and maternal severe infection pertain to the ITT populations of babies and women, respectively. The primary outcome of neonatal mortality pertains to liveborn neonates only, within the ITT population of babies. We also plan to conduct a secondary PP analysis using the PP population defined above. The main population for the analysis of secondary outcomes will also be the ITT population. This analysis will also be corrected for multiplicity.

Baseline characteristics will be compared between groups to detect imbalances in prognostic variables that could bias the results. However, given the large sample size for the trial, randomization is likely to prevent any such imbalance. Numbers and baseline characteristics of women lost to follow-up will also be compared between study groups to detect any imbalances. Most study outcomes are binary variables, for which the number of participants, number of missing values and percentages by group will be reported. The intervention arm will be compared against the control arm for the primary outcomes using risk ratios with 95% CIs. Risk differences and two-sided *p* values will also be reported. The statistical technique used to conduct tests and obtain confidence intervals will be a logistic model with a binomial distribution and the log link to obtain relative risks. The identity link will be used to obtain risk differences. The stratifying variable of study hospital will be included in the model.

For continuous variables, the number of participants, the number of missing values, minima, maxima, means and standard deviations or medians, quartiles and interquartile range (IQR) by group will be reported as appropriate. The intervention arm will be compared against the control arm using mean differences and 95% CIs. The statistical technique used to conduct tests and obtain confidence intervals for this type of variable will be a general linear model with an appropriate distribution and including centre in the model as a stratifying variable.

Planned stratified analyses include by subgroups of type of preterm birth (planned preterm birth versus other), gestational age at randomization, gestational age at first dose, multiple and singleton pregnancy, study site/country, hospital capacity level, interval from time of randomization to birth, mode of delivery, and use of tocolytics. All models will be fitted using SAS Software version 9.3 (SAS Institute Inc., Cary, NC, USA). A separate model will be fitted for each primary outcome. Results will be reported according to Consolidated Standards of Reporting Trials (CONSORT) guidelines [31].

### Trial monitoring

Monitoring activities will be conducted overall, per site and per hospital. WHO has prepared standard operating procedures for all monitoring activities. In-person monitoring visits to participating hospitals will be conducted by country investigators, WHO staff and external, independent clinical trial monitors. These visits will verify that the trial is being conducted according to the study protocol and manual of operations, including screening and informed consent procedures, storage and use of study intervention (i.e. ampoules remaining for each participant), data collection and management, and handling of any adverse events. The frequency and intensity of monitoring will reflect the rate of recruitment and site performance. In the event that any serious or urgent issues are identified, additional monitoring visits or checks will be implemented. The data management team will review per-hospital and per-site rates of recruitment, adverse events and serious adverse events, and other key progress indicators on a monthly basis.

A Data and Safety Monitoring Board (DSMB) has been appointed. The DSMB is made up of five members, including an independent Chair (maternal and newborn baby health epidemiologist), a statistician, and three technical experts (obstetrics/gynaecology, neonatology, bioethics) familiar with the intervention, maternal and newborn baby health care, and clinical trial methodology, with no important conflicts of interest. The DSMB will convene at least annually to review trial progress, as well as review findings of interim analyses (see below). The rates of preselected specific adverse outcomes of interest to the trial (safety outcomes) will be aggregated across treatment groups and reported to the DSMB on a monthly basis. A DSMB member’s review of unexpected adverse events on an ‘as-needed’ basis may be sought.

### Interim analyses and stopping rules

Three interim analyses are planned, based on potential relative reductions of 40%, 30% and 20% at the three time points, with evidence reaching the 0.001 *p* value as required for recommending early stopping according to the Haybittle-Peto rule (see below). Assuming a baseline outcome rate of 25%, these three interim analyses correspond with intervention group outcome rates of 15%, 17.5% and 20%. Allowing for 10% drop-out, the corresponding sample sizes are 816, 1491 and 3447; it was agreed that target sample sizes of 800, 1500 and 3500 for the three planned interim analyses will be used. These correspond to recruitment of 13%, 25% and 58% of the total sample size. Blinded interim analyses will be reviewed by the DSMB. The unblinded interim analyses will also be available to the DSMB statistician, in the event the DSMB decides to review unblinded interim analysis findings. The DMSB may request additional interim analyses, or reschedule interim analyses as needed.

The Haybittle-Peto stopping rule will be applied on the primary outcomes of neonatal death and any baby death. Using this rule, a two-sided test of hypothesis to assess superiority of one of the groups (intervention or placebo) will be conducted. If the result is significant at α = 0.001, the DSMB will consider recommending stopping the trial for superiority of one of the groups. Any recommendation to stop the trial after the results of an interim analysis will not be guided only by statistical considerations, but also by practical issues (adverse events, ease of treatment administration, unanticipated costs), as well as clinical considerations or external new information. The Trial Coordinating Unit (TCU) will be ultimately responsible for early stopping of the trial upon DSMB recommendations.

### Training

Prior to commencement of recruitment, study site research teams complete an in-person training workshop on study procedures, according to a standardized study manual of operations. This workshop emphasizes GCP standards, the need for accurate and thorough data reporting and vigilance in identifying, detecting and reporting any possible adverse events, safety concerns or protocol deviations. Country and hospital investigators will maintain a valid GCP certificate throughout the trial. Standardized training for relevant staff will also be conducted at study sites to optimize use of obstetric ultrasound for gestational age estimation, use of transcranial ultrasound for assessment of neonatal intraventricular haemorrhage, and essential care of preterm newborn babies.

### Trial oversight

The TCU comprises staff from two WHO departments (maternal and newborn babies) and the trial statistician. The principal investigators are experienced maternal and neonatal health researchers from participating sites. The trial steering group (TSG) is composed of the TCU, the principal investigators, a group of external technical advisors and an independent Chair. Members of the TSG do not have important conflicts of interest. Any major changes to the study protocol will be decided by the TSG, and communicated to the relevant ethical committees and trial registry.

### Ethical considerations

The trial protocol was reviewed and approved by the WHO Ethics Review Committee (ERC.0002851). All participating sites received ethics approval from the relevant IRBs (see Additional file 4), as well as the relevant national regulatory authorities. Annual reports on study progress will be provided to these committees. Informed consent will be obtained from all study participants prior to their participation by study staff. An international clinical trial insurance provider has been engaged to provide indemnity insurance for trial participants.

## Discussion

Preterm birth is an important global public health issue, affecting an estimated 15 million live births worldwide each year [1]. The safety and efficacy concerns surrounding use of ACS in hospitals in low- and middle-income countries have arrested efforts to scale-up the use of ACS in these settings.

This trial will address key knowledge gaps around efficacy and safety of ACS in low-resource countries, and strengthen the overall evidence base on the efficacy of ACS by doubling the number of participants in the Cochrane review on this topic [23].

This trial has several strengths. It will recruit participants across five low-resource countries in sub-Saharan Africa and south Asia and is powered for critical mortality outcomes of the baby and possible effects on infectious morbidity in women. No previous trial has been independently powered for neonatal mortality, and trials have generally not followed participants into the community to assess impacts on neonatal mortality to 28 days of life. The ACTION-I trial will use standard definitions of maternal and newborn baby outcomes to 28 completed days after birth, and standard collection procedures to measure these outcomes. The eligibility criteria are not overly selective, ensuring that results will generalize to women at risk of imminent preterm birth. The hospitals have been carefully selected to reflect the minimum requirements for maternal care of women at risk of imminent preterm birth, as well as adequate care of preterm newborn babies. It is thus anticipated that the findings will be generalizable to other, similar hospitals in low- to middle-income countries.

Results of this trial will be published in a peer-reviewed, open-access journal by the WHO ACTION Trials Collaboration. The trial will also inform the update of the evidence base supporting WHO recommendations on ACS use, national and local clinical guidelines, and policies on ACS use. Currently corticosteroids are widely available, and dexamethasone sodium phosphate (4 mg/mL) is already listed on the WHO Model Essential Medicines List for use in preterm birth [28]. Hence, we anticipate that if this trial demonstrates efficacy, access to ACS should not face the challenges of a new drug or formulation.

## Trial status

Version 1.10 (6 February 2018) commenced recruiting 24 December 2017. The expected date to complete recruitment is October 2020.

## Additional files

Additional file 1: SPIRIT 2013 checklist: recommended items to address in a clinical trial protocol and related documents. (DOC 126 kb)

Additional file 2: ACTION-I trial: primary and secondary outcomes. (DOCX 21 kb)

Additional file 3: ACTION I trial informed consent form, v1.2, 7 February 2017. (PDF 199 kb)

Additional file 4: List of Institutional Review Boards who have approved the ACTION-I trial. (DOCX 18 kb)

## Data Availability

Not available.

## Abbreviations

ACS: Antenatal corticosteroids
ACT: Antenatal Corticosteroids Trial
ACTION: Antenatal Corticosteroids for Improving Outcomes in preterm Newborns
DSMB: Data and Safety Monitoring Board
GCP: Good clinical practice
IQR: Interquartile range
IRB: Institutional Review Board
ITT: Intention-to-treat
PP: Per-protocol
SMS: Short message service
SPIRIT: Standard Protocol Items: Recommendations for Interventional Trials
TCU: Trial Coordinating Unit
TSG: Trial Steering Group
WHO: World Health Organization

## Footnotes

1 “Birth planned or expected within 48 h” is considered as imminent preterm birth, and includes: a) a woman presents with signs of spontaneous preterm labour, and the obstetric care physician assesses the likelihood of birth in the next 48 h to be high; or b) spontaneous prelabour rupture of membranes (PPROM) is confirmed; or c) a decision has been made to end pregnancy within 48 hours to improve maternal and/or neonatal outcomes through labour induction or caesarean section on the basis of any medical or obstetric (fetal and maternal) indications.

2 WHO currently recommends that women should receive an ultrasound during pregnancy prior to 24 weeks. In order to be eligible, the gestational age assessment must be informed by an antenatal obstetric ultrasound. Participating hospitals all have obstetric ultrasound available, and will determine gestational age based on a standard algorithm.

3 Women are only eligible if there are no clinical signs of severe, acute infection as per an assessment by an obstetric care physician. Clinical or laboratory features of severe infection could include (but are not limited to) maternal fever ≥ 38.0 °C, maternal and/or fetal tachycardia, purulent or foul smelling vaginal discharge, uterine tenderness, maternal leucocytosis and/or bacterial culture indicative of infection.
