# Supplementary material for: The World Health Organization ACTION-I (Antenatal CorTicosteroids for Improving Outcomes in preterm Newborns) Trial: a multi-country, multi-centre, two-arm, parallel, double-blind, placebo-controlled, individually randomized trial of antenatal corticosteroids for women at risk of imminent birth in the early preterm period in hospitals in low-resource countries
Source: Trials. 2019 Aug 16;20:507. doi: 10.1186/s13063-019-3488-z (PMC6698040; doi:10.1186/s13063-019-3488-z)
Supplement: Supplementary file 2 — ACTION-I trial: primary and secondary outcomes. (DOCX 21 kb) [file 13063_2019_3488_MOESM2_ESM.docx]

Additional file 2. ACTION-I Trial: Primary and secondary outcomes

| **PRIMARY OUTCOMES:** | **OPERATIONAL DEFINITION AND MEASUREMENT** |
| --- | --- |
| 1. Neonatal death | Death of a live birth by 28 completed days of life. |
| 1. Any baby death | Any death of a fetus (post randomization) or death of a live birth within 28 completed days of life |
| 1. Possible maternal bacterial infection | Occurrence of maternal fever or clinically suspected or confirmed infection, for which therapeutic antibiotics were used  *Captured during hospital admission/s only* |
| ***A. For the neonate*** | |
| **A1. Mortality outcomes** | |
| 1. Stillbirth | Any death of a fetus (post randomization) |
| 1. Early neonatal death | Death of a live birth by 7 completed days of life |
| **A2. Morbidity outcomes** | |
| 1. Severe respiratory distress* | Clinical features are the presence of fast breathing (respiratory rate ≥ 70 breaths per minute) AND at least one of the following clinical signs:  1. Marked nasal flaring during inspiration,  2. Expiratory grunting audible with naked ear  3. Severe chest in drawing.  AND  SpO2 less than 90%, or use of supplemental oxygen |
| 1. Neonatal sepsis* | Defined as the presence of at least two (or more) of the following signs:   - Stopped feeding well - Severe chest in-drawing - Fever (body temperature of 38 °C or greater) - Hypothermia (body temperature less than 35.5 °C) - Movement only when stimulated or no movement at all - Convulsions |
| 1. Severe Intraventricular haemorrhage (sIVH) | Defined as a Papile’s intraventricular hemorrhage classification grade 3 or 4, as per transcranial ultrasound assessment  Liveborn neonates <34 weeks at birth will be routinely screened with transcranial ultrasound. Liveborn neonates ≥ 34 weeks at birth will receive transcranial ultrasound if indicated.  Transcranial ultrasound assessment will be performed at day 7 postnatal or discharge (if discharge occurs before 7 days after birth). |
| 1. Neonatal hypoglycaemia* | Neonatal hypoglycemia is defined as any blood glucose measure less than 45 mg/dl (2.6mmol/l)  All liveborn newborns in hospital will have glucose levels recorded at 6 and 36 hours (before feeding or IV fluids). Any documented hypoglycaemia will also be recorded. |
| 1. Apgar score at 5 minutes | Assessment of neonatal vitality at 5 minutes after birth. Reported as Apgar score, and proportion of babies with Agar <7 |
| ***B. For the Woman*** | |
| **B1. Mortality outcomes** | |
| 1. Maternal death | Any maternal death in a trial participant, from time of randomization to 28 completed days postpartum |
| **B2. Morbidity outcomes** | |
| 1. Maternal fever | Maternal fever ≥38.0 C since randomization (on any one occasion, during hospital admission/s only) |
| 1. Chorioamnionitis | Chorioamnionitis (suspected or confirmed) based on clinical assessment by obstetric care physician  Clinical or laboratory features may include:   - - Maternal fever ≥38.0 C   - Maternal and/or fetal tachycardia   - Purulent or foul smelling vaginal discharge   - Uterine tenderness   - Maternal leukocytosis   - Bacterial culture indicative of infection   measured during hospital admission/s only |
| 1. Postpartum endometritis | Postpartum endometritis (suspected or confirmed) based on clinical assessment by obstetric care physician  Clinical or laboratory features may include:   - - Maternal fever ≥38.0 C   - Maternal and/or fetal tachycardia   - Purulent or foul smelling vaginal discharge   - Uterine tenderness   - Maternal leukocytosis   - Bacterial culture indicative of infection   measured during hospital admission/s only |
| 1. Wound infection | Infection of a wound or incision site (including perineal tear, episiotomy incision or CS abdominal incision), suspected or confirmed by obstetric care physician  Measured during hospital admission/s only |
| 1. Non-obstetric infection | Acute non-obstetric infection (suspected or confirmed) based on clinical assessment by obstetric care physician.  This includes:   - respiratory tract infection (including pneumonia, pharyngitis, sinusitis or similar) - Urinary tract infection - Pyelonephritis - Acute cholecystitis   *Malaria is specifically excluded from this outcome*  Measured during hospital admission/s only |
| ***C. Process of care outcomes*** | |
| **C1. Measures of care given to neonate** | |
| 1. Major neonatal resuscitation at birth | The use of positive pressure ventilation for more than one minute |
| 1. Timing of breast milk feeding initiation* | Timing of initiation of breast milk feeding in hours after birth (breastfeeding, cup or tube feeding). |
| 1. Time to full enteral feeding* | Timing to full enteral feeding (in days) |
| 1. Use of oxygen therapy* | Defined as any use of oxygen therapy, using any method |
| 1. Length of oxygen therapy* | This is defined as the total number of days of oxygen use during hospital stay. The total number of days will be counted, even if use was intermittent. |
| 1. Use of continuous positive airway pressure (CPAP) ventilation* | Defined as any use of CPAP during admission to neonatal special care unit/ward |
| 1. Length of use of continuous positive airway pressure (CPAP) ventilation* | Total number of days used will be counted, even if use is interrupted for hours or days. |
| 1. Use of mechanical ventilation (MV)* | Any use of MV during admission |
| 1. Length of use of mechanical ventilation (MV)* | Total number of days used will be counted, even if use is interrupted or intermittent |
| 1. Any use of parenteral therapeutic antibiotic therapy* | Any use of therapeutic antibiotics (intravenous or intramuscular) for 5 or more days |
| 1. Length of use of parenteral therapeutic antibiotic therapy* | Total number of days of use of parenteral antibiotic therapy |
| 1. Use of surfactant treatment* | Any use of surfactant |
| 1. Number of doses of surfactant treatment* | Total number of doses of surfactant treatment |
| **C2. Health service utilization (newborn)** | |
| 1. Length of hospital stay after birth | Length of stay in hospital after birth in complete days (initial postnatal hospitalization only) |
| 1. Admission to a special care unit (SCU) | Admission to special neonatal care unit or neonatal intensive care unit after birth (initial postnatal hospitalization only) |
| 1. Length of admission to special care unit (days) | Length of admission to SNCU or NICU in days |
| 1. Newborn readmission for health care at facility | Any readmission to a health care at facility, for any reason. |
| 1. Length of stay for newborn readmission | Length of readmission stay in facility in days |
| 1. Number of newborn readmission for health care at facility | Number of readmissions for health care at facility, for any reason. |
| 1. Cause of neonatal readmission for health care at facility | All causes of neonatal readmission to health care at facilities will be recorded as per clinical diagnosis |
| **C3. Measures of care given to woman** | |
| 1. Therapeutic antibiotics | Therapeutic antibiotics for suspected or confirmed infection (obstetric or non-obstetric).  *Use of antibiotics for prophylaxis is not included in this outcome.*  Measured during hospital admission/s only |
| 1. Number of days of therapeutic antibiotic use | Number of days of use of therapeutic antibiotics for suspected or confirmed infection (obstetric or non-obstetric).  Use of antibiotics for prophylaxis is not included in this outcome.  Measured during hospital admission/s only |
| 1. Any antibiotic use | Any use of antibiotics in a randomized participant while in facility (prophylactic or therapeutic)  Measured during hospital admission/s only |
| **C3. Health service utilization (woman)** | |
| 1. Length of total maternal hospitalization for birth (days) | number of days which women are hospitalized for birth (i.e. the admission in which birth occurs).  Measured from day of admission to day of official discharge from facility, in days |
| 1. Any postpartum maternal readmission to facility | Any postpartum readmission of the woman to hospital for any reason up to 28 completed days postpartum |
| 1. Number of maternal readmissions to facility | Number of postpartum readmissions of the woman to hospital for any reason up to 28 completed days postpartum |
| 1. Cause of maternal readmission to facility | All causes of maternal readmission to hospital will be recorded as per clinical diagnosis |
| 1. Any referral of woman to another facility for treatment of complications | Any referral of woman to another hospital for treatment of complications |
| ***Measures of compliance*** | |
| 1. Compliance with study allocation | Defined as the proportion of women who complete the entire course, as per the allocation |
| 1. Use of repeat course | Total number and proportion of women who received a repeat course of ACS or placebo |
| 1. Total number of ACS doses received | Total number of ACS doses received (initial and repeat) |
| 1. Time from initiation of first dose until birth | Defined as the time from initiation of first dose (ACS or placebo) to birth, measured in hours |

* Measured during initial postnatal hospitalization only, until death, discharge or completed day 7 (whichever comes first)
