# Supplementary material for: The World Health Organization ACTION-I (Antenatal CorTicosteroids for Improving Outcomes in preterm Newborns) Trial: a multi-country, multi-centre, two-arm, parallel, double-blind, placebo-controlled, individually randomized trial of antenatal corticosteroids for women at risk of imminent birth in the early preterm period in hospitals in low-resource countries
Source: Trials. 2019 Aug 16;20:507. doi: 10.1186/s13063-019-3488-z (PMC6698040; doi:10.1186/s13063-019-3488-z)
Supplement: Supplementary file 3 — ACTION I trial informed consent form, v1.2, 7 February 2017. (PDF 199 kb) [file 13063_2019_3488_MOESM3_ESM.pdf]

[SITE INSTITUTIONAL LETTERHEAD]

Informed Consent Form (ICF)

This ICF is for women attending participating hospitals in [**COUNTRY**], and who we are inviting to participate in research on use of antenatal corticosteroids for women at risk of imminent preterm birth.

The title of our research project is "A65913: A multi-country, multi-centre, two-arm, parallel, double-blind, placebo-controlled, randomized trial of antenatal corticosteroids for women at risk of imminent birth in the early preterm period in hospitals in low-resource countries to improve newborn outcomes."

[Name of Principal Investigator]
[Name of Organization]
SPONSOR: World Health Organization
[Name of Proposal and version]

This Informed Consent Form has two parts:

- **Information Sheet (to share information about the research with you)**
- **Certificate of Consent (for signatures if you agree to take part)**

You will be given a copy of the full Informed Consent Form

PART I: Information Sheet

Introduction
Good day, I am […….], working for [……..]. We are doing a research project on preterm birth, which is a common condition affecting pregnant women. I am going to give you information and invite you to be part of this research project. You do not have to decide right now whether or not you will participate in the research. Before you decide, you can talk to anyone you feel comfortable with about the research.

There may be some words that you do not understand. Please ask me to stop as we go through the information and I will take time to explain. If you have questions, you can ask them of me, the research doctor or the staff.

Purpose of the research

Preterm birth (when a baby is born too early in pregnancy, for example before 37 weeks) is a common condition. It affects around 10% of pregnancies. In the past, when a woman was at high risk of giving birth preterm, doctors have given the mother an injection of steroids. This injection can speed up the development of the baby's lungs, so that when they are born they have a better chance of survival. The injection can also prevent or reduce some illnesses that affect preterm newborns.

The steroid injection is widely used in many advanced countries. It is also used in countries like ours although not to the same extent as in advanced countries. When it is used in higher-level hospitals in high-income countries with adequate healthcare resources to take care of preterm babies and their mothers it appears to beneficial to the survival of preterm babies. However, when the same steroid injection is used on a large scale in the community, clinics and primary and referral level hospitals in African or Asian countries, it is not helpful and can even be harmful to the newborn and the mother.

We do not know if this injection is helpful when used in our country, in a hospital like this one, where we have moderate quality of care for mothers and preterm babies. There is no research that tells us that there is a good basis for a choice between using and not using the steroid injection in this hospital.

This research will try to establish whether these injections are safe and effective for mothers and preterm babies when used in hospitals like this one.

Type of Research Intervention

This research will involve some injections into your muscle, usually in your arm. Up to four injections may be given. If for some reason you do not give birth during this admission and you go home, you may also receive some more injections when you come back to hospital.

Participant selection

We are inviting all pregnant women who are at risk of preterm birth and who attend this hospital to participate in this research.

- ***Questions to ensure understanding:*** *Do you know why we are asking you to take part in this study? Do you know what the study is about?*

Voluntary Participation

Your participation in this research is entirely voluntary. It is your choice whether to participate or not. Whether you choose to participate or not, all the services you receive at this hospital will continue and nothing will change.

If you choose not to participate in this research project, you will be offered the treatment that is routinely offered in this hospital. You may change your mind and stop participating, even if you agreed earlier.

- ***Questions to ensure understanding:*** *If you decide not to take part in this research study, do you know what your options are? Do you know that you do not have to take part in this research study, if you do not wish to? Do you have any questions?*

Information on the Trial Drug Dexamethasone

The drug we are testing is called dexamethasone. It is used in humans for many different illnesses, and is also commonly used in pregnant women. We now want to test dexamethasone on people who are giving birth preterm in this hospital.

Some participants in the research will not be given dexamethasone. Instead, they will be given an injection of water that is sterile. There is no risk associated with this injection and no known problems.

Procedures and Protocol

A. Unfamiliar Procedures

Because we do not know if dexamethasone is better than sterile water in this setting, we need to compare the two. To do this, we will put people taking part in this research into two groups. The groups are selected by chance, as if by tossing a coin.

Participants in one group will be given the test drug while participants in the other group will be given the sterile water, called a placebo. It is important that neither you nor we know which of the two drugs you are given. When the research is finished, we will then compare which of the two has the best results.

The healthcare workers will be looking after you and the other participants carefully during the study. If we are concerned about what the drug is doing, we will find out which drug you are getting and make changes. If there is anything you are concerned about or that is bothering you about the research, please talk to me or one of the other researchers.

You and your baby will otherwise receive the normal treatment for your condition, according to national guidelines. This means that you will be closely looked after during labour and delivery. It is possible that you and your baby may receive some tests if they are needed. This can include taking a sample of blood. If this is needed, we will take blood from your arm using a syringe and needle. The amount of blood taken will depend on what tests need to be done. If there is any leftover blood sample it will be destroyed.

We will also take a drop of your baby's blood using a heel prick. This is a routine neonatal test, where a small prick is made in your baby's heel and the drop of blood put onto a piece of card. This will allow us to conduct further tests of your baby's blood.

If any further tests are performed, such as blood tests, X-rays or other tests, our researchers will record the results in our files.

B. Description of the Process

If you agree to participate, you will receive either the test drug or the dummy/pretend drug.

- During your stay in hospital for childbirth, one of our researchers will collect information on the health of you and your baby.
- After you leave hospital, we would like to contact you by phone or a visit to see how your and your baby's health are doing.
- If you or your baby becomes unwell after you go home, you may need to come back to hospital. If so, we would like you to come back to this hospital. We will also collect information on the health of you and your baby during that admission (if it occurs).
- After 7 days and 28 days have passed, we would like to visit you at your home to ask you some questions about yours and your baby's health.

Duration

The research takes place during your childbirth and up to 28 days after the birth. During that time, it will be necessary for us to contact you by SMS or telephone, and for a researcher to visit you after 7 days and 28 days have passed. Once the last visit is completed, the research is over.

In the future (for example, some years after the birth) researchers may wish to contact you to see how you and your baby are doing. We would also like permission to contact you in the future, so we can follow up on your and your baby's health.

- ***Questions to ensure understanding:*** *Do you know how many days after birth we would like to visit you at your home? Do you have any other questions? Do you want me to go through the procedures again?*

Side Effects

In this trial, half the participants will receive a small dose of steroids for a short period of time. This drug can very rarely cause side effects. Any side effects are temporary, and will end when the treatment is finished. You may experience difficulty sleeping or headache due to the treatment, however these symptoms can occur often during pregnancy and childbirth.

The injection can cause some temporary swelling or soreness around the place where the injection goes into your arm.

We will follow you closely and keep track of any problems. We may use some other medicines to decrease the symptoms of the side effects or reactions. Or we may stop the use of one or more medicines. If this is necessary, we will discuss it together with you and you will always be consulted before we move to the next step.

Risks

Being born preterm increases the risk that your baby will have illnesses in early life, and is at increased risk of death. In this research project, there is a risk that your baby's health will not be improved by the medicine we are testing (i.e. that the medicine is not effective).

While the risk of these occurring is low, you should be aware of the possibility. We will try to decrease the chances of this event occurring, but if something unexpected happens, we will ensure you are informed and get the best available care.

- ***Questions to ensure understanding:*** *Do you understand that you may have some unwanted side-effects from the medicines? Do you understand that participating in this study may carry some risks? Do you have any other questions?*

Benefits

There may not be any direct benefit for you in this research, but your participation is likely to help us find the answer to whether this drug is effective or not. If the medicine is proven to be effective, we aim to help other people like you benefit from it.

Reimbursements

You will not be given any other money or gifts to take part in this research. If any participant (woman or her baby) become ill or have injuries as a result of being in this study, they will be given immediate treatment for the illness or injuries at [**HOSPITAL**] according to the hospital's / Ministry of Health's guidelines, at no cost.

Insurance

All participants in this trial are covered by a clinical trial insurance policy, in the event of harm occurring as a direct result of a study medication or procedure that was used in this study.

- ***Questions to ensure understanding:*** *Can you tell me if you have understood correctly the benefits that you will have if you take part in the study? Do you know if the study will pay you for participating or not? Do you have any other questions?*

Confidentiality
With this research, it is possible that if others in the community are aware that you are participating, and they may ask you questions. We will not be sharing the identity of those participating in the research.

The information that we collect from this research project will be kept confidential. Information about you that will be collected during the research will be put away, and no-one but the researchers will be able to see it. Any information about you will have a number on it instead of your name. Only the researchers will know what your number is and we will lock that information up with a lock and key. It will not be shared with or given to anyone else, except researchers at the World Health Organization.

- ***Questions to ensure understanding:*** *Did you understand the procedures that we will be using to make sure that any information that we as researchers collect about you will remain confidential? Do you have any questions about them?*

Sharing the Results
Your confidential information will not be shared. Once the research is completed, we will publish the results in order that other interested people may learn from our research and apply what we have learned. We will also make the data from this research available to other researchers, so more can be learnt about this drug. All information about you will be anonymous – it will not contain your name or any personal identifying information.

Right to Refuse or Withdraw
You do not have to take part in this research if you do not wish to do so. Refusing to participate will not affect your treatment at this hospital in any way. You may stop participating in the research at any time that you wish without losing any of your rights as a patient here.

Alternatives to participating, and what care would you get or may be available elsewhere if you do not take part in the study

If you do not wish to take part in the research, you will still be provided with the established standard treatment available at this hospital. That means you are able to give birth at this hospital and will receive the same care that other women like you normally receive. The final decision on whether you will receive the steroid injection would be taken by you after discussing with the doctors that are taking care of you. You can discuss this freely with them.

You are also free to go to another hospital, or be referred to another hospital, for your birth if you wish. At that hospital, the final decision on whether you will receive the steroid injection would be taken by you after discussing with the doctors that are taking care of you. You can discuss this freely with them.

Who to Contact
If you have any questions you may ask them now or later, even after the study has started. If you wish to ask questions later, you may contact any of the following:

[name]
[address/telephone number/e-mail]

This proposal has been reviewed and approved by [name of the local IRB], which is a committee whose task it is to make sure that research participants are protected from harm. If you wish to find about more about the IRB, contact [name, address, telephone number.]). It has also been reviewed by the Ethics Review Committee of the World Health Organization (WHO), which is funding/sponsoring/supporting the study.

- ***Questions to ensure understanding:*** *Do you know that you do not have to take part in this study if you do not wish to? Do you know that you can ask me questions later, if you wish to? Do you know that I have given the contact details of the person who can give you more information about the study?*

You can ask me any more questions about any part of the research study, if you wish to. Do you have any questions?

PART II: Certificate of Consent

I have read the foregoing information, or it has been read to me. I have had the opportunity to ask questions about it and any questions that I have asked have been answered to my satisfaction. I consent voluntarily to participate as a participant in this research.

I also agree to be contacted in the future after this research is completed, for longer-term follow up research.

Print Name of Participant__________________

Signature of Participant ___________________

Date ___________________________
Day/month/year

If illiterate
A literate witness must sign (if possible, this person should be selected by the participant and should have no connection to the research team). Participants who are illiterate should include their thumb-print as well.

I have witnessed the accurate reading of the consent form to the potential participant, and the individual has had the opportunity to ask questions. I confirm that the individual has given consent freely.

Print name of witness_____________________ AND Thumb print of participant

Signature of witness ______________________

Date ________________________
Day/month/year

Statement by the researcher/person taking consent
I have accurately read out the information sheet to the potential participant, and to the best of my ability made sure that the participant understands that the following will be done:
1.
2.
3.

I confirm that the participant was given an opportunity to ask questions about the study, and all the questions asked by the participant have been answered correctly and

to the best of my ability. I confirm that the individual has not been coerced into giving consent, and the consent has been given freely and voluntarily.

A copy of this ICF has been provided to the participant.

Print Name of Researcher/person taking the consent________________________

Signature of Researcher /person taking the consent__________________________

Date ___________________________
Day/month/year
