# Supplementary material for: The World Health Organization ACTION-I (Antenatal CorTicosteroids for Improving Outcomes in preterm Newborns) Trial: a multi-country, multi-centre, two-arm, parallel, double-blind, placebo-controlled, individually randomized trial of antenatal corticosteroids for women at risk of imminent birth in the early preterm period in hospitals in low-resource countries
Source: Trials. 2019 Aug 16;20:507. doi: 10.1186/s13063-019-3488-z (PMC6698040; doi:10.1186/s13063-019-3488-z)
Supplement: Supplementary file 4 — List of Institutional Review Boards who have approved the ACTION-I trial. (DOCX 18 kb) [file 13063_2019_3488_MOESM4_ESM.docx]

**Appendix 2. List of Institutional Review Boards who have approved the ACTION-I Trial**

| **Site** | **Institutional Review Board** | **Identification number** | **Date of approval** |
| --- | --- | --- | --- |
| Bangladesh | Bangabandhu Sheikh Mujib Medical University Institutional Review Board | BSMMU/2017/8002 | 30/7/2017 |
|  | Johns Hopkins School of Public Health Institutional Review Board | IRB No: 00007684 | 2/8/2017 |
|  | Bangladesh Medical Research Council National Research Ethics Committee | BMRC/NREC/2016-2019/264 | 21/6/2017 |
| India^[[1]](#footnote-1)^ | S Nijalingappa Medical College Institutional Ethics Committee on Human Subjects Research | SNMC/IECHSR/2016-17/A-55/1.0 | 30/3/2017 |
|  | B. L. D. E University Institutional Ethical Committee, Bijapur | BLDEU/EC/2016-17/200 | 15/3/2017 |
|  | KLE University Ethics Committee, Belgaum | KLEU/EC/2016-17/D-3981 | 27/02/2017 |
|  | Indian Council of Medical Research | 5/7/41/2017-RBMH | 27/7/2017 |
| Kenya | Kenyatta National Hospital – University of Nairobi Ethics Review Committee | P45/01/2017 | 11/10/2017 |
|  | Coast General Hospital Ethics Review Committee | ERC-CGH/MSc/VOL.I/37 | 16/2/2018 |
| Nigeria (Ibadan) | University of Ibadan/University College Hospital Ethics Committee, Ibadan | UI/EC/17/0055  UI/EC/17/0056  UI/EC/17/0057 | 25/5/2017 |
|  | Federal Capital Territory Health Research Ethics Committee | FHREC/2017/01/18/10-3-17 | 22/5/2017 |
|  | Lagos State University Teaching Hospital Health Research and Ethics Committee | LREC 06/10/794  LREC 06/10/795 | 14/3/2017 |
| Nigeria (Ile-Ife) | University of Abuja Teaching Hospital Human Research Ethics Committee, Abuja, Nigeria | FCT/UWATH/HREC/PR/045 | 2/5/2017 |
|  | Obafemi Awolowo University Teaching Hospitals Complex Ethics and Research Committee | IRB/IEC/0004553 | 15/03/2017 |
|  | University of Benin Teaching Hospital Ethics Research Committee | ADM/E 22/A/VOL. VII/1483 | 17/5/2017 |
|  | University of Ilorin Teaching Hospital Ethics Research Committee | UITH/CAT/189/19B/228 | 5/5/2017 |
|  | Ondo State Health Research Ethics Committee | AD/4693 Vol II/86 | 21/4/2017 |
|  | Sacred Heart Hospital Ethical Committee | SHH/EC/EA/009/04/17 | 21/4/2017 |
| Pakistan | Aga Khan University Ethics Review Committee | 4724-Ped-ERC-17 | 21/6/2017 |

1. The ACTION-1 Trial was also registered with the Clinical Trial Registry of India (CTRI/2017/04/008326) [↑](#footnote-ref-1)
